# Interferon signaling predicts response to oncolytic virotherapy

**DOI:** 10.18632/oncotarget.26679

**Published:** 2019-02-22

**Authors:** Cheyne Kurokawa, Evanthia Galanis

**Affiliations:** Department of Molecular Medicine, Mayo Clinic, Rochester, MN, USA; Division of Medical Oncology, Mayo Clinic, Rochester, MN, USA

**Keywords:** oncolytic therapy, measles, inteferon signaling, permissiveness

Oncolytic virotherapy is emerging as a promising therapeutic approach for cancer treatment and involves the use of viruses that selectively replicate and kill tumor cells. Results from clinical trials clearly indicate that not all patients achieve a favorable therapeutic response, however [1]. Talimogene laherparepvec (TVEC), an HSV-1 strain expressing GMCSF was approved in 2015 by FDA and later the European Medicines Agency for the treatment of metastatic melanoma marking a significant breakthrough in the field of oncolytic virotherapy. In the phase III trial that led to FDA approval, a durable objective response was observed in 16% of patients treated with TVEC as compared to 2% of patients treated with GM-CSF alone [2]. Although this study met its primary endpoint of durable objective response leading to the approval of TVEC for this indication, the therapeutic benefit from TVEC was still only achieved in a subset of treated patients enrolled in the trial. Similarly, efficacy has been observed in subsets of patients treated with other oncolytic viruses, such as vaccinia virus, poliovirus, replication competent retroviral vectors, vesicular stomatitis virus, the oncolytic adenovirus Delta-24-RDG and measles virus (MV) [1, 3-5]. While the benefit that individual patients treated with oncolytic virotherapy achieve can be impressive, a relatively small percentage of patients appear to derive such benefit. Therefore, an in-depth analysis to examine differences among responders and non-responders is required to better understand the mechanism of response and enrich oncolytic virotherapy trials with patients more likely to derive benefit.

Oncolytic MV is currently being investigated in clinical trials for several tumor types, such as recurrent glioblastoma (GBM), ovarian cancer, breast cancer, mesothelioma, and multiple myeloma. In a recently completed phase I trial of MV for patients with recurrent glioblastoma, we studied the extent of viral replication in tumor samples resected five days after administration of the first viral dose, when in accordance with preclinical data maximum replication was expected. Despite similar eligibility criteria for all study patients, we observed that the extent of virus replication in tumors varied greatly: from undetectable to 6 × 10^7^ genome copies/μg of RNA [6]. Since levels of viral receptor expression are thought to be a key factor accounting for the variability in patient responses to oncolytic virotherapy, we initially hypothesized that expression levels of the three MV receptors (Nectin-4, CD46 and SLAM) could explain the observed difference in replication among our patients [7]. To our surprise analysis of expression levels of the three MV receptors revealed comparable levels among study patients, thus suggesting that a post-entry restriction mechanism rather than an entry related mechanism was responsible for the observed differences in replication [6]. In order to investigate this further we studied gene expression differences in primary GBM patient-derived xenografts (PDXs) that were permissive or resistant to MV infection and cell killing. A comparison of differentially activated pathways between MV resistant and permissive cells revealed a pre-existing antiviral state in resistant cells, characterized by the constitutive activation of the antiviral interferon (IFN) pathway. This allowed us to develop a diagonal linear analysis algorithm (DLDA), a weighted gene signature consisting of 22 interferon stimulated genes (ISG). This DLDA algorithm was prospectively validated in 35 patient derived GBM xenografts and 86 ovarian cancer avatars and was shown to be predictive across tumor types; importantly, it could also predict and explain the differences in viral replication observed in our trial of MV in recurrent GBM patients. This represents the first example of a molecular algorithm that can predict clinical responses to oncolytic virotherapy: patients with a DLDA score <-250 had wide-spread viral replication; patients with a DLDA score >150 had no viral replication, while patients with DLDA scores between 150 and -250 had intermediate levels of viral replication. The extent of virus replication was inversely correlated with the level of IFN activation (ρ=-0.717; p-value 0.03). These results provide important insights that can impact the design of oncolytic virotherapy vectors and clinical trials. First, our findings demonstrate that tumor cells can have intact IFN signaling that can effectively restrict the replication of oncolytic viruses. This challenges the previous perception that IFN signaling, which is part of the host's innate immune response against potential pathogens, is predominantly impaired in tumors [8]. During the initial design of many oncolytic virus platforms, several groups hypothesized that a defective IFN system in tumor cells would always allow for tumor specific replication of the virus. Therefore, oncolytic viruses were attenuated to disrupt the virus' ability to counter the IFN system, thus allowing for the selective replication in tumor cells. Several viruses currently being tested in clinical trials, such as MV, VSV and Herpes Simplex virus 1, have mutations and/or deletions in their genome to impair the virus' ability to counter the IFN system [1]. These mutations could impair the ability of the virus to replicate in tumors with active IFN signaling and impact clinical efficacy.

Furthermore, we demonstrated that the pre-existing antiviral state can be reversed by blocking JAK/STAT signaling for example through the use of the FDA approved JAK1/2 inhibitor, Ruxolitinib. Combining MV with Ruxolitinib resulted in more than 100-fold increase in virus production in resistant lines. [6]. In addition to its impact on viral replication, signaling through the JAK/STAT pathway is important for activation of the immune system: as recently demonstrated by *Benci et al.*, resistance to checkpoint inhibitors can be driven by persistent IFN signaling [9]. By carefully considering timing and sequence of JAK/STAT inhibitor administration when combined with virotherapy, JAK inhibition could play a dual role by both promoting virus replication, as well as inhibiting an important pathway for resistance to immunotherapy approaches. Of note, preclinical data support that the synergy between virotherapy with immune checkpoint inhibitors, such as antibodies targeting PD-1 or CTLA-4 can be observed even in the context of limited viral replication [10]. In this context, patients whose tumors are predicted to allow intermediate levels of viral replication (DLDA scores between -250 and 150) could still represent excellent candidates to receive MV in combination with immune checkpoint inhibitors

Although our algorithm was tested in MV treated patients and xenografts, active IFN signaling is expected to impact replication and efficacy across many other oncolytic platforms and assessment of its predictive value is warranted. For example, our data indicate that approximately 15 percent of GBM patients are expected to be very permissive to viral replication because of very low expression levels of ISG. Response rates in contemporary GBM trials employing other oncolytic platforms are very similar for example 12% in patients treated with Delta-24-RGD or 13 % of patients treated with the replicating retrovirus Toca-511 in phase I trials [3, 5] raising the possibility that IFN signaling represents a determinant of response across other oncolytic platforms as well. Prospective ongoing validation of our ISG based predictive signature in clinical trials and enrichment of future studies with patients more likely to respond to virotherapy can allow us to optimize on the therapeutic benefit of these approaches.

## References

[1] Lawler SE (2017). JAMA Oncol.

[2] Andtbacka RH (2015). J Clin Oncol.

[3] Cloughesy TF (2016). Sci Transl Med.

[4] Galanis E (2015). Cancer Res.

[5] Lang FF (2018). J Clin Oncol.

[6] Kurokawa C (2018). J Natl Cancer Inst.

[7] Liu TC (2007). Cancer Res.

[8] Stojdl DF (2000). Nat Med.

[9] Benci JL (2016). Cell.

[10] Hardcastle J (2017). Neuro Oncol.

